# Changes in Fatty Acid Composition of Human Milk in Response to Cold-Like Symptoms in the Lactating Mother and Infant

**DOI:** 10.3390/nu9091034

**Published:** 2017-09-19

**Authors:** Andrew S. Gardner, Ibrahim A. Rahman, Ching T. Lai, Anna Hepworth, Naomi Trengove, Peter E. Hartmann, Donna T. Geddes

**Affiliations:** 1 School of Molecular Sciences, The University of Western Australia, Crawley, Perth, WA 6009, Australia; brahim.hjrahman@moh.gov.bn (I.A.R.); Ching-tat.lai@uwa.edu.au (C.T.L.); anna.hepworth@uwa.edu.au (A.H.); peter.hartmann@uwa.edu.au (P.E.H.); Donna.Geddes@uwa.edu.au (D.T.G.); 2 School of Health and Sciences, The University of Notre Dame Australia, 32 Mouat Street, Fremantle, WA 6959, Australia; naomi.trengove@nd.edu.au

**Keywords:** human milk, breastfeeding, fatty acids

## Abstract

Infants rely on their innate immune systems to protect them from infection. Human milk (HM) contains fatty acids (FAs) and monoacylglycerols that are known to exhibit antiviral and antibacterial properties in vitro. The specific fat content of HM may potentially affect the efficacy of this antimicrobial activity. This preliminary study investigates whether the proportions of FA in HM change in response to infections, leading to cold-like symptoms in the mother or infant. Milk samples were obtained from mothers (*n* = 26) when they and their infants were healthy, and when mother, infant, or both suffered cold-like symptoms. The milk was hydrolysed and FA proportions were measured using gas chromatography. Fifteen FAs were recorded, of which eight were detected in sufficient quantities for statistical analysis. The proportions of capric (C10:0) and lauric acids (C12:0) in HM were significantly lower, and palmitic acid (C16:0) was higher when mothers and infants were ill compared to healthy samples. Palmitoleic (C16:1, n-7) and stearic acid (C18:0) proportions were higher in HM when the infant was unwell, but were not related to maternal health. Whilst the differences detected were small (less than 0.5%), the effects may be additive and potentially have a protective function. The value of further studies is certainly indicated.

## 1. Introduction

Human milk provides the ideal nutrition for an infant during the first six months of life, leading to optimal growth and development [1,2]. The link between infant health and breastfeeding has long been noted, and has more recently been the subject of intensive research [3]. Breastfeeding has been associated with a decreased incidence of many infections, including necrotizing enterocolitis, gastroenteritis, severe respiratory illness, otitis media, and urinary tract infections [2].

At birth, the infant immune system displays several immaturities. The acquired immune system is naïve, needing time to develop immunological responses and memory. This causes infants to be reliant on aspects of the innate immune system to protect them from serious infections. Human milk provides immune protection from the mother in the form of antibodies, particularly in the secretory immunoglobulin A (sIgA) fraction. Human milk is also known to contain multiple bioactive factors that form part of the innate immune system of the gastrointestinal tract [2]. These include proteins such as lactoferrin and lysozyme, oligosaccharides, glycoproteins, and lipids. The lipids, one of the major nutrients in milk, are provided in the form of milk fat globules composed of a core of triacylglycerols surrounded by a membrane of phospholipids, cholesterol, glycoproteins, and proteins [4]. The core triacylglycerols are not protective until hydrolysis releases their free fatty acids (FAs) and monoacylglycerol products. In the infant, this hydrolysis occurs through the action of lingual and gastric lipases which can penetrate the membrane of the milk fat globules [4].

The antimicrobial activity afforded by these fatty acids varies considerably depending on chain length, degree of unsaturation, and presence of reactive groups [5], with lauric (C 12:0, dodecanoic) and linoleic (C18:2, 9,12-octadecadienoic) acids reported to be among the most potent [6]. They appear to work via a detergent-like action on lipid-coated microorganisms [6]. It is thought that these FAs are incorporated into the lipid layer, where they cause instability of the lipid membrane, which results in the rupture of the lipid envelope and death of the microorganism [7,8]. These free FAs and monoacylglycerols may therefore provide the breast-fed infant with protection from enteric microorganisms, prior to maturation of the infant immune system [3].

Various in vitro studies have shown the protective properties of FAs against lipid-coated microorganisms [7,9,10] including respiratory syncytial virus (RSV), herpes simplex virus type 1 (HSV-1), and group B streptococcus [6,7,9,10]. The concentration of antimicrobial lipids in the infants’ stomach one hour after feeding is such that even a ten-fold dilution results in minimal reduction of activity against enveloped viruses [10]. Given the dynamic nature of milk production and its important role in protecting the infant from infections, the concentration of antimicrobial agents in breastmilk, including FAs, might conceivably be altered in response to infections.

During lactation, the mammary gland becomes the primary site for de novo FAs synthesis [11,12]. However, the presence of a mammary-specific thioesterase II limits the chain length to 14 and thus the medium chain FAs (MCFAs) such as capric, lauric, and myristic acids are known as de novo FAs [13]. In contrast, most of the long chains FAs (LCFAs), such as palmitic acid, are derived from the diet [12,13,14,15,16,17,18,19]. Even though they could be synthesised by the action of elongases and desaturases on MCFAs, most are absorbed from the maternal circulation and are therefore referred to as preformed FAs. Some LCFAs, such as linoleic and α-linolenic acid, cannot be synthesised and are obtained solely from the diet and body stores, and are referred to as essential FAs. In this study, both arachidonic (AA) and docosahexaenoic acid (DHA) were grouped as conditionally essential n3 and n6 polyunsaturated fatty acids (PUFAs) respectively. Even though syntheses of both FAs from their precursors are possible, the process is slow compared to the uptake of preformed FAs and may be compromised by nutritional deficiencies as well as inflammatory conditions [20].

Fatty acids are also known to enhance the ability of some proteins to bind to lipid membranes by increasing their hydrophobicity. For example, the attachment of palmitic acid to haemagglutinin (palmitoylation) of influenza A virus facilitates the attachment of the virus to the target cell and thus replication of the virus [21]. Similarly, it has also been reported that the bonding of myristic acid to the structural protein (myristoylation) of the human rhinovirus is necessary for the attachment of the virus to the target cell membrane and for the replication of that virus [22]. Therefore, in contrast to their potential protective role, some fatty acids might increase the transmission or severity of these infections.

This study aimed to establish whether a cold-like infection in either the mother, her infant, or both would trigger a response by the mammary glands to alter the proportions of specific FAs in breastmilk. If such changes are beneficial, we hypothesise that lauric and linoleic FA proportions in breastmilk may be increased in line with their antimicrobial action, while the proportions of palmitic and myristic acids may decrease due to their roles in enhancing viral replication.

## 2. Materials and Methods 

Twenty-six breastfeeding women residing within the metropolitan area of Perth, Western Australia were recruited for the study and provided with a study package, including consent and demographics forms. Mothers were excluded if they were unlikely to continue breastfeeding or were unlikely to remain within the metropolitan area during the study period. Ethics approval for this study was given by the Human Research Ethics Committee of the University of Western Australia RA/4/1/2130.

Each sample set involved collecting 2–5 mL of breastmilk expressed from each breast at approximately the same time of day for three consecutive days (*n* = 6 samples). The first set of three milk samples from each breast was collected during the first three days of the study. Then, if the mother or infant showed cold-like symptoms, another set of samples were collected following the same protocol for three consecutive days. Three weeks after the cold-like symptoms disappeared, the last set of samples were collected following the same protocol for three consecutive days. Thus, 18 milk samples were collected when either mother, infant, or both suffered cold-like symptoms during the study period. Mothers who remained healthy and had healthy infants throughout the study period were asked to collect a second set of samples at the end of the study period (six months), bringing their total number of milk samples to 12. The mothers initially stored the milk samples in domestic freezers at −20 °C. The samples were collected from the mothers within a week and then stored at −80 °C until required for analysis.

The definitions of cold-like symptoms used in this study were: a body temperature outside the normal range (36.5–37.5 °C); an infection affecting the upper respiratory tract and characterised by a sore throat, blocked and running nose; general tiredness with a duration of 3 to 5 days. A mother or an infant who had at least two of these symptoms was considered to display cold-like symptoms.

FA analysis was carried out using gas chromatography. Samples were run in duplicates. Total lipids were extracted using chloroform:methanol (2:1 *v*/*v*) and the extracts evaporated to dryness under nitrogen. FAs were then trans-esterified using 1% H_2_SO_4_ in methanol for 2 hours at 70 °C. After cooling, the resulting methyl esters were extracted into n-heptane and transferred to vials containing NaHCO_3_ as a dehydrating agent. FA methyl esters (FAME) were then separated and quantified using a Shidmadzu 2010 gas chromatograph equipped with a 50-mm capillary column (0.32-mm internal diameter) coated with BPX-70 (0.25-µm film thickness; SGE Pty Ltd., Ringwood, VIC, Australia). Each sample (1 µL) was then injected in to the column using an automatic injector (Shimadzu AOC 20i, Shimadzu Corporation, Kyoto, Japan) at a split ratio of 20:1. The injector temperature was set at 250 °C with the detector (flame ionisation) temperature at 300 °C. The initial oven temperature was 140 °C. The temperature was initially programmed to rise to 155 °C at 5 °C/min, then slowly to 165 °C at 1 °C/min, then back at 5 °C/min to 230 °C, and finally at 10 °C/min to 250 °C, where it was held for 5 minutes. Helium was used as the carrier gas at a velocity of 35.0 cm/min. Identification of FA peaks was made by comparing their retention times to that of known FAME standards (Sigma–Aldrich, St Louis, MO, USA). The recovery of FA C17:0 (added as heptadecanoylglycerol to milk prior to extraction) with respect to the addition of FA C15:0 (added as pure methyl pentadecanoate after transesterification) was 97.5% with a coefficient of variation of 4.0% (*n* = 10). Individual FA peaks were quantified as percentages of the total area under the FA peaks and reported as a percentage of total FAs.

Analysis of data was undertaken using R 2.9.0 (R Development Core Team 2009). Packages nlme and lattice were used for linear mixed models and lattice plots, respectively. *p* < 0.05 was considered significant. Replicates were compared by testing for significant differences between models with random effects of sample or replicates within sample. As no differences were seen (*p* > 0.99), replicates values were averaged for subsequent analysis. Where only one value was available, that was considered the measured value. For each of the FAs of interest, the main explanatory variables of the models considered were infant age, maternal health, infant health, and dyad health. Other explanatory variables considered were gestational age, birth weight, and maternal age. The random effect of the models was infant age and mother.

## 3. Results

### 3.1. Participant Characteristics

The mean age of the mothers was 34.8 years (range 28.0–44.0). The mean gestational age of the infants was 39.5 weeks (range 37.0–42.0) and the mean birth weight was 3.5 kg (range 2.9–4.2). The infant age at the time of recruitment was on average 311 days (range 13–1001), where most infants were aged less than 366 days (*n* = 22 infants), and just four infants were aged over 366 days. Seven of the infants received solid food as well as HM, and two received some formula to complement HM. Of the 26 mothers-infant dyads recruited, 23 suffered cold-like symptoms and collected appropriate samples, while three did not develop symptoms. Of the 23 dyads with cold-like symptoms, in 19 cases both mother and infant had symptoms. In one case, the mother had a second episode of cold-like symptoms, and collected additional samples (*n* = 8 samples). The other four mothers had cold-like symptoms, but their infants remained well throughout the study.

### 3.2. Fatty Acids 

Fifteen FAs were detected (Table 1) beginning with Caprylic (8:0) to the LC-PUFA DHA (22:6 n-3). The proportions of each FA were highly variable between samples. Only eight FAs (capric, lauric, myristic, palmitic, palmitoleic, stearic, oleic, and linoleic acids) were detected in sufficient quantities in all samples for robust statistical analysis.

### 3.3. Effect of the Health of Mother, Infant, or both on FA Proportions in Milk

The proportions of capric and lauric acid were significantly lower in the breastmilk of mothers when the mother, infant, or both displayed cold-like symptoms (Table 2). The proportion of palmitic acid was higher in breastmilk when the mother was unwell, but was not related to infant health. Palmitoleic and stearic acid proportions were lower in breastmilk when the infant displayed cold-like symptoms, but were not related to maternal health. No relationships were found between proportions of myristic, oleic, and linoleic acids and health status.

## 4. Discussion

This study suggests that cold-like infections in mother or infant or both may result in partial suppression of the synthesis of capric and lauric acids, both de novo FAs, accompanied by an increase in palmitic acid, indicating a response by the mammary gland. However, the magnitudes of the differences in relative amounts for capric and lauric acids, 0.04–0.05% and 0.29–0.31%, respectively, were small. Given its demonstrated antimicrobial activity [6], a reduction in the proportion of lauric acid in unwell mothers was not predicted.

A reduction of lauric acid in response to infant infection is consistent with a study that demonstrated an increased risk of mother to child transmission of HIV with a 1% higher concentration of lauric acid and a decreased risk with higher concentrations of n6 LC-PUFAs in breastmilk, including dihomo-γ-linolenic (DGLA) and arachidonic acid (AA) [23]. Further, HIV transmission was directly proportional to the concentration of lauric acid, despite its known antiviral action in vitro. The decrease in lauric acid concentrations in the present study, in response to infant and maternal cold infection, was only a third (0.30%) of that reported in mothers with HIV compared to healthy mothers. HIV is a chronic infection and therefore may have a more sustained effect on the mammary gland, whereas in our study the cold-like infection was acute and only sustained for a short period. We found no differences in the LC-PUFAs detected in this study between the well and cold samples, suggesting that maternal diet did not change significantly during that period. Given the decreased risk of transmission of HIV to the infant with higher levels of LC-PUFAs, it is conceivable that raising levels during lactation via the diet may reduce cold-like infections in breastfed infants. Whilst the changes in lauric acid noted in our study were small, the decreased mean proportions support the concept that the mammary gland can respond to cold-like infections by suppressing de novo FA synthesis, with the possibility of limiting transmission of the pathogen from mother to infant.

During a respiratory infection in either the mother or infant, a reduction in the concentration of palmitic and myristic acids in the breastmilk may conceivably be beneficial to the infant through reducing the binding ability of the virus to the target cell membrane. However, we found an increase in palmitic acid during maternal and dyad infection, suggesting an active role of palmitic acid. This would not support a beneficial protective effect for the infant. An inverse relationship between the concentration of de novo FAs and palmitic acid has previously been reported in term milk, which could account for the observed increase in the proportion of palmitic acid when the mother is ill. The decreased synthesis of de novo FAs may have been compensated for by an increased uptake of preformed FAs such as palmitic acid [20].

It was hypothesised that the mean proportion of linoleic acid would be increased following maternal cold infection due to its in vitro antimicrobial properties and to reduce the risk of transmission of the infection to the infant, in line with the observations of Villamor et al. [23], who reported that the risk of transmission of HIV was inversely proportional to the concentration of DGLA and arachidonic acid (AA) [23]. All three n6 PUFAs; linoleic, DGLA and AA, were shown to have similar protective potency in vitro [10]. However, this protective effect of n6 PUFAs was not found by Badiou et al. [24]. We did not find a statistically significant relationship between the mean proportion of the essential n6 PUFA linoleic acid and the health of the mother, infant, or both.

The small size of the sample collection in this study is a limitation. When analysed individually, one mother had significant differences in the mean proportion for all eight selected FAs; capric, lauric, myristic, palmitic, palmitoleic, stearic, and linoleic acid, between cold and well samples. This mother had two cases of cold symptoms which allowed a greater sample number (*n* = 38 samples). Another limitation of the study is the focus on cold-like symptoms. As the common cold Rhinovirus is not enveloped, it may be less susceptible to the surfactant-like antimicrobial action of FAs. Responses to enteral infections in mother and infant would be of interest in this regard. Further limitation of the study are that the diet, fasting state, dehydration, and insulin levels of the mothers were not controlled.

This is the first study that examines the relationship between changes in the relative amounts of FAs in breast milk in response to a clinical infection, such as a cold. While this study does not attempt to identify the mechanism by which these FA proportions were altered, the results nevertheless indicate that there are significant changes in FA proportions associated with an infection. Isaac et al. [10] reported that the antimicrobial effects of fatty acids from milk work additively and synergistically with milk peptides, rather than through the proportion of individual FAs [10]. Hence, even though the changes observed in the concentrations of individual FAs were small, the changes could potentially be physiologically significant, and the value of further studies is certainly indicated.

## Figures and Tables

**Table 1:** Overall percentage proportions for fatty acids detected in the milk of mothers when they and their infants were healthy and when they and their infants were suffering cold-like symptoms. Data are presented as a mean percentage (±SE) and range.

Group	FAs	Mean % of Total FAs (±SE) by Weight	Range
De novo FAs	Caprylic (C8:0)	0.14 (±0.1)	0.06–0.28
	Capric (C10:0)	1.13 (±0.3)	0.40–2.19
	Lauric (C12:0)	5.90 (±0.8)	1.76–11.85
	Myristic (C14:0)	7.87 (±1.0)	2.29–17.00
Preformed FAs	Palmitic (C16:0)	23.86 (±0.5)	17.26–30.91
	Palmitoleic (C16:1 n-7)	2.29 (±0.4)	1.06–4.56
	Stearic (C18:0)	7.39 (±0.6)	3.91–12.68
	Oleic (C18:1 n-9)	35.79 (±0.7)	25.73–52.11
	Arachidic (C20:0)	0.49 (±0.2)	0.23–0.85
	Gadoleic (C20:1 n-9)	0.30 (±0.1)	0.18–0.48
	Dihomo-γ-linolenic (C20:3 n-6)	0.25 (±0.1)	0.14–0.25
Essential FAs	Linoleic (C18:2 n-6)	10.90 (±0.9)	5.41–23.95
	α- linolenic (C:18:3 n-3)	1.00 (±0.4)	0.45–2.35
Conditionally essential FAs	Arachidonic (C20:4 n-6)	0.35 (±0.1)	0.21–0.54
	DHA (C22:6 n-3)	0.36 (±0.3)	0.14–0.98

**Table 2:** Significance of the relationship between the health predictor and the fatty acid proportion. Where significant, the direction and magnitude of the change is included.

Fatty Acid	Maternal Health	Infant Health	Dyad Health
Capric acid	*p* = 0.014 0.04% lower in unwell	*p* = 0.006 0.05% lower in unwell	*p* = 0.004 0.05% lower in unwell
Lauric acid	*p* = 0.008 0.30% lower in unwell	*p* = 0.020 0.29% lower in unwell	*p* = 0.006 0.31% lower in unwell
Myristic acid	*p* = 0.824	*p* = 0.996	*p* = 0.891
Palmitic acid	*p* = 0.008 0.45% higher in unwell	*p* = 0.154	*p* = 0.004 0.49% higher in unwell
Palmitoleic acid	*p* = 0.087	*p* = 0.045 0.10% lower in unwell	*p* = 0.215
Stearic acid	*p* = 0.476	0.001 0.41% lower in unwell	*p* = 0.516
Oleic acid	*p* = 0.285	*p* = 0.758	*p* = 0.450
Linoleic acid	*p* = 0.672	*p* = 0.080	*p* = 0.867
